# Evolution of petal patterning: blooming floral diversity at the microscale

**DOI:** 10.1111/nph.70370

**Published:** 2025-07-08

**Authors:** Erin Doody, Edwige Moyroud

**Affiliations:** ^1^ Sainsbury Laboratory Cambridge University Cambridge CB2 1LR UK; ^2^ Department of Genetics University of Cambridge Downing Street Cambridge CB2 3EH UK

**Keywords:** cell type, corolla, evo‐devo, floral development, natural variation, novelty, petal patterning, pollinator attraction

## Abstract

The flowers of angiosperms are extraordinarily diverse. While most floral variation is visible to the naked eye, this diversity goes beyond the macroscale: Floral organs comprise an underappreciated range of cell types that generate a multitude of patterns across their surfaces and give rise to novel structures. Because diverse cell patterns provide adaptations to biotic and abiotic factors, they also contribute to angiosperm evolution and speciation. Yet, how such diversity originates remains to be understood. In this review, we focus on petals, which together form the corolla, to examine the mechanisms patterning floral surfaces at the cellular level. We summarize current research aiming to understand how cell fate specification and controlled cell growth (proliferation and expansion) are achieved with high spatial resolution during petal development. We also examine the adaptive potential for such patterns and how they contribute to plant fitness and diversification. Finally, we discuss promising directions for future research on the evolution of petal patterning at the microscale and identify outstanding questions that technological advances now make it possible to address.

**Table:** 

	Content	
	Summary	2538
I.	Introduction	2538
II.	Currently available model species provide diverse systems to understand petal evo‐devo at the microscale	2539
III.	Cell patterns on petal surface as adaptive traits	2540
IV.	Organizing and modifying the colour, shape and texture of petal pavement cells	2542
V.	Diversifying petals by changing cell type specification: the case of floral trichomes	2547
VI.	Patterning cell behaviour supports the evolution of novel petal features	2549
VII.	Drivers of natural selection on petal patterns and their contribution to speciation	2550
VIII.	Conclusion and insights	2551
	Acknowledgements	2552
	References	2552

## I. Introduction

Flowers – the reproductive structures of angiosperms – exhibit an extraordinary diversity of forms across the plant kingdom. A typical flower is comprised of male and female reproductive organs (stamens and carpel or pistil) surrounded by sterile parts: the petals, collectively known as the corolla, and an outer whorl of sepals, together forming the calyx (Endress, 2001). These structures are as functional as they are beautiful: A properly formed flower is vital for reproductive success of the plant. By facilitating self‐fertilization, wind pollination or pollinator attraction, floral morphology has a direct impact on reproduction and contributes to reproductive isolation and speciation (Barrett, 2010). As a result, the genetic mechanisms and adaptive significance of flower diversity provide an excellent framework for understanding the evolution and development of angiosperms.

Floral organs often vary in number, shape, symmetry or arrangement and the mechanisms accounting for the evolution of those traits have been extensively reviewed elsewhere (Litt & Kramer, 2010; Bowman *et al*., 2012; Bowman & Moyroud, 2024; Khojayori *et al*., 2024). However, many species produce highly specialized petals with complex shapes, lobed or fringed margins or the addition of various appendages. Such intricate petals are widespread, yet little is known about how these complex morphologies are created during development (Endress & Matthews, 2006).

Petal surfaces also harbour diversity at the microscopic scale, displaying a variety of cell types across their epidermis. The spatial distribution of different cell types is genetically encoded, allowing for the construction of robust patterns during development. As petal primordia emerge from the floral meristem, epidermal cells grow (proliferate and expand) and differentiate, acquiring specific colour, shapes and texture. Genetic modifications to petal cell behaviour during development yields morphological variation that can in turn contribute to the emergence of diverse patterns of cellular features or even specialized petal structures, such as nectar spurs (Moyroud & Glover, 2017). Therefore, exploring the mechanisms regulating cell behaviour across the corolla not only sheds light on the evolutionary processes accounting for the diversification of angiosperms, it also provides a framework to understand crosstalk between patterning and morphogenesis, two core developmental processes and their contribution to biodiversity.

In this review, we outline recent research on the mechanisms underlying the development of complex floral morphologies at the microscale, focusing on variation of petal traits between and within species that influence the evolution of flowering plants. Because petals constitute a major interface between a plant and its environment, cellular arrangements across the petal epidermis fulfil important adaptive functions, such as attracting pollinators and protecting reproductive organs in harsh environments. We highlight outstanding questions for understanding the mechanisms patterning the petal surface, and how emerging model organisms and novel technologies are being leveraged to understand petal evolution at the cellular level and revolutionize the field of petal evo‐devo. We discuss the genetic mechanisms that orchestrate cell differentiation to create patterns across the petal, by modifying epidermal cell features or specifying distinct cell types, and how these processes are selected upon to create novel patterns. We examine how the regulation of cell growth can be modified during development to create elaborate petal structures, such as nectar spurs, and how these principles can impact the evolution of novelty. Finally, we also outline recent studies that improve our understanding of petal patterns evolution in natural populations, arguing it is essential to investigate petal patterning across diverse species and accessions in the field.

## II. Currently available model species provide diverse systems to understand petal evo‐devo at the microscale

**Fig. 1:**
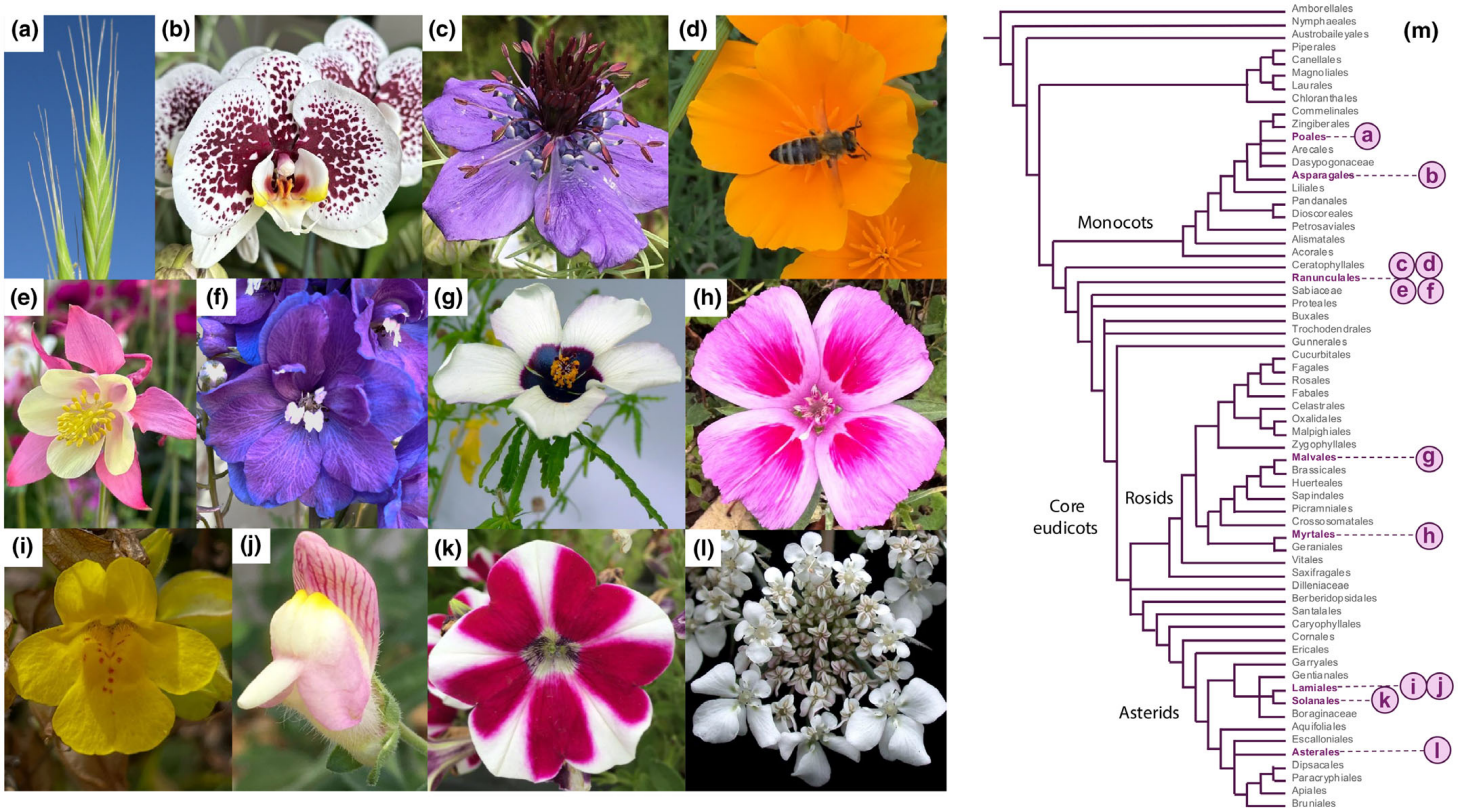
Model systems currently used to study flower evo‐devo at the microscale. (a) *Brachypodium distachyon*, (b) Phalaenopsis moth orchid, (c) *Nigella* sp., (d) *Eschscholzia californica*, (e) *Aquilegia coerulea* sp., (f) *Delphinium* sp., (g) *Hibiscus trionum*, (h) *Clarkia amoena*, (i) *Mimulus guttatus*, (j) *Antirrhinum majus*, (k) *Petunia × hybrida* and (l) *Daucus carota*. (m) Simplified phylogenetic tree of the angiosperms according to the APGIV classification (The Angiosperm Phylogeny Group *et al*., 2016), depicting the distribution of these model species. Pictures in (a, l) are credited to Laval University and Harry Rose, respectively, via Wikimedia Commons.

Research in *Arabidopsis thaliana* largely established the basis of what is known about angiosperm development. However, *A. thaliana* depends predominantly on self‐pollination and its flowers are simple and small, making it difficult to answer questions about the evolution of complex flowers (Vallejo‐Marín & Barrett, 2009). As a result, model systems, such as *Antirrhinum*, *Petunia* and *Mimulus* (Fig. 1; Table 1), became foundational species for understanding petal development and evolution, and are now being used to study the role of flower morphology in natural selection and speciation (Schwarz‐Sommer *et al*., 2003; Yuan, 2019).

**Table 1:** Examples of current model systems suitable to study flower evo‐devo at the microscale.

	Gene manipulation	Resources available	Petal cellular trait studied	References	Model system review
Phalaenopsis (Moth orchid)	VIGS, stable transformation (tissue culture)	Genome, transcriptome, diverse species and accessions	Pigmentation, cell shape, cuticle texture, scent, organ and elaboration	Lu *et al*. (2022) Hsu *et al*. (2021) Liao *et al*. (2020) Pramanik *et al*. (2020) Mao *et al*. (2024)	Tsai *et al*. (2008) Zhang *et al*. (2023)
Nigella	VIGS	Chloroplast genome, transcriptome and inter‐ and intraspecies variation	Pigmentation, pseudonectaries and cell shape	Yuan *et al*. (2023) Yao *et al*. (2019) Liao *et al*. (2020) Zhang *et al*. (2020)	Damerval & Becker (2017)
Eschscholzia (California poppy)	VIGS	Draft genome and transcriptome	Pigmentation and cell shape	Wilts *et al*. (2018) Pollack *et al*. (2019)	Becker *et al*. (2023)
Aquilegia (columbine)	VIGS	Genome, transcriptome, inter‐ and intraspecific variation and mutant collection	Nectar spur and nectary development	Cabin *et al*. (2022) Edwards *et al*. (2022)	Kramer (2009) Kramer & Hodges (2010) Yant *et al*. (2015) von Balthazar *et al*. (2025)
Delphinium	VIGS	Chloroplast genome, transcriptome and natural interspecific variation	Organ morphology, cell shape, pigment and nectar spur development	Zhang *et al*. (2024)	Samarah *et al*. (2020)
Hibiscus	Stable transformation (tissue culture)	Annotated genome, transcriptome, inter‐ and intraspecific variation and CRISPR/Cas9 gene editing	Pigmentation, cuticle texture and cell shape	Moyroud *et al*. (2022) Riglet *et al*. (2024) Yao *et al*. (2019)	
Clarkia	VIGS	Genome draft and natural intraspecific variation	Pigmentation and scent	Lin & Rausher (2021a, 2021b)
Mimulus (monkeyflower)	Stable transformation (floral dipping)	Annotated genome, transcriptomes, collection of mutants and natural accessions, inter‐ and intraspecific variation and CRISPR/Cas9 gene editing	Pigmentation (biosynthesis of pigments and complex pattern formation), corolla tube formation and flower symmetry	Liang *et al*. (2023) Gao *et al*. (2024) Zhang *et al*. (2021)	Yuan (2019)
Antirrhinum (snapdragon)	NA	Genome, transcriptomes, mutant collection, inter‐ and intraspecific variation and horticultural varieties	Pigmentation and cell shape	Durán‐Castillo *et al*. (2021) Tavares *et al*. (2018)	Davies *et al*. (2018) Schwarz‐Sommer *et al*. (2003) Mizzotti *et al*. (2014)
Petunia	Stable transformation (tissue culture)	Genome, transcriptome, mutant collection, inter‐ and intraspecific variation, horticultural varieties and CRISPR/Cas9 gene editing	Pigmentation (visible & UV), cell shape and petal cuticle synthesis	Chopy *et al*. (2023) Skaliter *et al*. (2023) Li *et al*. (2022)	Vandenbussche *et al*. (2016) Strazzer *et al*. (2023)
*Daucus carota* (wild carrot)	Stable transformation (tissue culture)	Genome, transcriptome, natural accessions and CRISPR/Cas9 gene editing	Pigmentation and complex floral architecture	Duan *et al*. (2024) Baczyński *et al*. (2022)	Que *et al*. (2019)
*Brachypodium distachyon*	Stable transformation (tissue culture)	Annotated genome, transcriptome, collection of mutants and natural accessions, inbred lines and CRISPR/Cas9 gene editing	Lodicule, lemma and glume development	Patterson *et al*. (2024)	Schrager‐Lavelle *et al*. (2017) Raissig & Woods (2022) Kellogg (2015)

VIGS, Virus‐Induced Gene Silencing.

The establishment of an increasingly expanding range of diverse model organisms (Fig. 1; Table 1) is allowing for the transition from descriptive studies of floral patterns to more mechanistic understanding of complex pattern modification and the evolutionary consequences of such changes. Species recently developed as models with more complex floral structures, including *Aquilegia*, *Hibiscus* and *Nigella*, open new directions by helping to understand how and why elaborate corollas evolve (Kramer & Hodges, 2010; Zhang *et al*., 2020; Cabin *et al*., 2022; Raissig & Woods, 2022; Cheng *et al*., 2023; Yuan *et al*., 2023; Riglet *et al*., 2024). Monocot models also offer promising research avenues. According to the floral quartet model, members of the MADS‐box gene family code for transcription factors that combine with each other to form distinct tetrameric complexes (MADS‐box complexes) that specify different floral organ identities (Theissen & Saedler, 2001; Bowman & Moyroud, 2024). Interestingly, recent functional investigations in orchids have uncovered that distinct MADS‐box complexes also specify sophisticated morphologies and pigmentation patterns across the perianth of Phalaenopsis species (Hsu *et al*., 2015, 2021; Pramanik *et al*., 2020). Grasses lack petals but have analogous organs called lodicules and genetic models, such as *Brachypodium*, are attractive systems to investigate whether those species deploy similar programmes to eudicots to pattern the surface of their floral organs (Schrager‐Lavelle *et al*., 2017). As transformation protocols are developed for an increasing number of organisms, we can now dissect the cellular mechanisms of more complex petal patterns and elucidate the genetic basis of floral traits that are not found in traditional models.

Additionally, due to the decreasing cost of whole‐genome sequencing and *de novo* assembly, studies using population genomics and genome scans are becoming more feasible in nonmodel systems. This is allowing for a number of recent studies that take advantage of naturally occurring genetic variation *in situ* to understand population genomics, hybridization and natural selection as it occurs in real time (Bradley *et al*., 2025; Field *et al*., 2025; Richardson *et al*., 2025). These resources can also assist in mapping complex quantitative traits with fine resolution, using quantitative trait locus (QTL) mapping and genome‐wide association studies. For example, a study in sunflowers used natural accessions to dissect the genetic basis of ultraviolet (UV) pattern diversity (Todesco *et al*., 2022). Together, these highlight a need for the collection of accessions in other species to encompass a wide genetic and phenotypic diversity at the cellular level. Such resources would likely illuminate petal evo‐devo at the microscale, echoing the contributions the 1001 Genomes project has made to our understanding of adaptation and evolution in *A. thaliana* (Alonso‐Blanco *et al*., 2016).

## III. Cell patterns on the petal surface as adaptive traits

Patterns created on the petal epidermis are ecologically relevant because they mediate biotic and abiotic interactions. For example, corolla appearance is important for attracting pollinating animals and has long been credited for the rapid diversification of angiosperms as it can lead to reproductive isolation and speciation (Van der Niet *et al*., 2014). A number of recent studies have begun to reveal novel functions for these patterns and markedly advance our understanding of their biological significance.

**Fig. 2:**
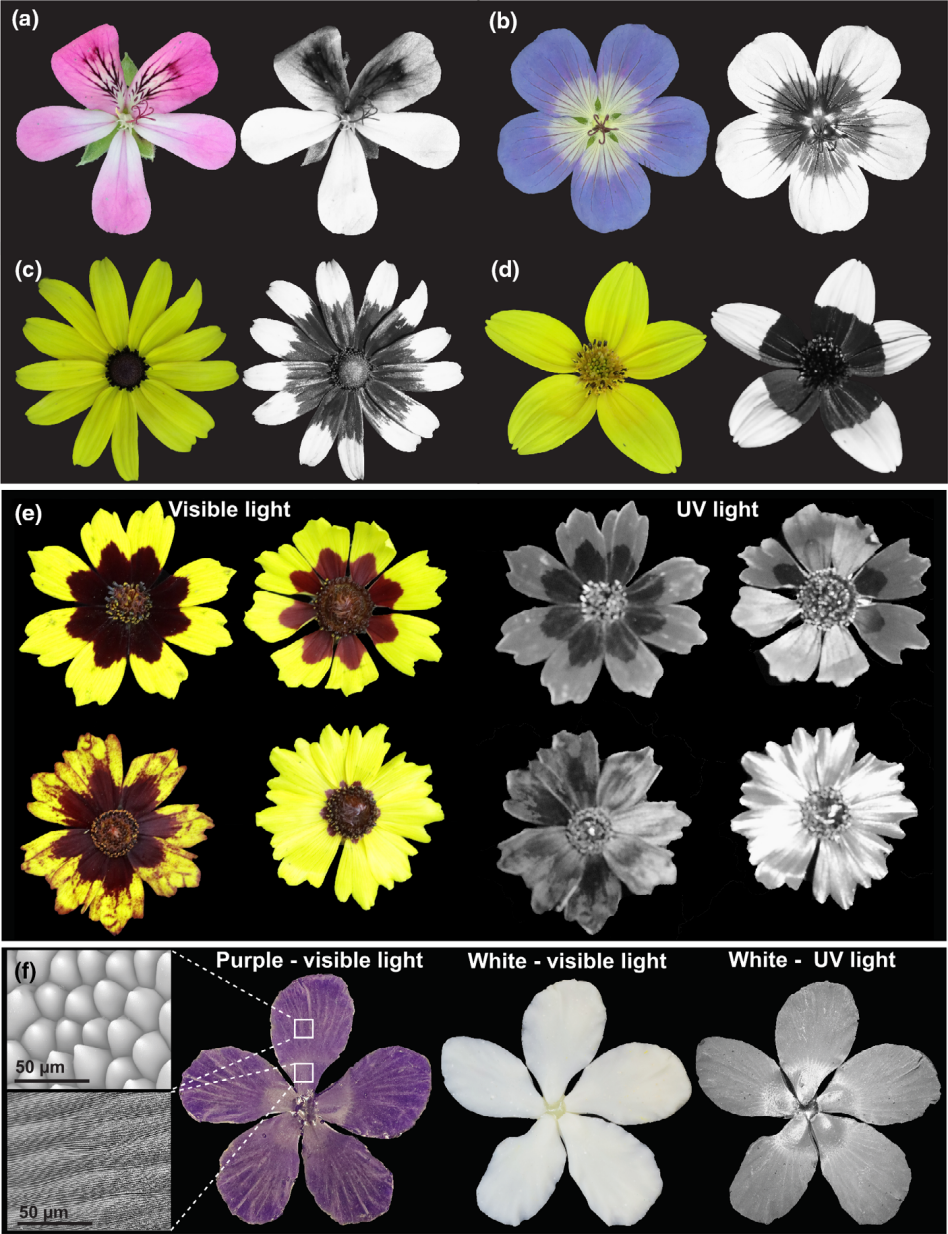
Differences in ultraviolet (UV) light reflecting and absorbing pigments as well as cell shape and texture across the petal surface can generate petal patterns in the UV range. (a–d) Images of flowers in human‐visible and UV light spectrums of (a) *Pelargonium* ‘Pink capitatum*’* (Geraniaceae), (b) *Geranium sylvaticum* (Wood Cranes‐Bill, Geraniaceae), (c) *Rudbeckia laciniata* (Cut‐leaf coneflower, Asteraceae) and (d) *Bidens triplinervia* (Asteraceae), each with variation in UV‐absorbing or reflective properties across the corolla. (e) Natural variation in petal pigmentation patterns and UV‐reflective properties of *Coreopsis tunctoria* (Nuttall Weed, Asteraceae), with variation in UV reflection varying with both pigment and cell texture. (f) Artificial epoxy resin flowers created by imprinting the petal of *Hibiscus trionum* in dental wax to replicate its surface: striated flat elongated cells in the proximal portion and smooth conical cells in the distal portion of the petal, as seen under SEM imaging (Top left: SEM image of distal smooth conical cells; Bottom left; SEM image of proximal flat striated cells). In the purple artificial flower (left), the conical cells create a mat velvety appearance on the corolla periphery while the flat striated cells render its centre shiny. This ‘structural’ bullseye is not easily visible to the human eye in the white artificial flower (middle) but become apparent when imaged with a UV camera (right), showing that pigment, shape and texture of epidermal cells all influence visible and UV light reflective properties of the petal.

Petal epidermal patterns are best known for their role in pollinator attraction. These patterns can enhance visibility of a flower from a distance or act as guides to direct insects to nectar or pollen after landing (Free, 1970; Whitney & Glover, 2007; Ojeda *et al*., 2016; Costa *et al*., 2017). Some of these motifs are not always perceptible by the human eye, such as patterns in UV‐absorbing or reflecting properties across the petal that often correlate with local pollinator vision (Fig. 2). For example, the gain of UV reflection in the petals of red poppies as they spread across Europe coincides with a shift in pollination system from beetles to bees (Martínez‐Harms *et al*., 2020). The presence of UV patterns on the corolla of many red flowers in the Mediterranean basin is proposed to be adaptations for pollination by hymenopterans (red‐blind insects) in environments where bird‐mediated pollination is not present (León‐Osper & Narbona, 2022). Motifs on the corolla can also lure pollinators by resembling food supply (Ma *et al*., 2016; Lunau *et al*., 2024) or brooding sites by mimicking the fungi fruiting bodies on which some species of flies lay their eggs (de Melo *et al*., 2011; Johnson & Schiestl, 2016; Abrahamczyk *et al*., 2021). In orchids and daisies, petal patterns also participate in sexual deception by emulating a pollinator's mating partners (Ellis & Johnson, 2009; Gaskett, 2011).

Petal patterns can also protect reproductive organs from stress, including heat, UV exposure, drought and herbivory. Larger UV bullseye patterns in silverweed and sunflower populations are believed to protect pollen from damage in environments with high levels of UV exposure (high altitude, artic and hot climates) or allow flowers to better resist desiccation (Koski & Ashman, 2015; Koski *et al*., 2020; Todesco *et al*., 2022). However, such a role might be species‐specific, as populations of *Clarkia unguiculata* exposed to lower levels of solar radiation produce larger, not smaller, UV‐absorbing bullseyes, suggesting that they do not provide significant pollen protection against UV rays (Peach *et al*., 2020). This advocates for similar studies to be conducted in a broader range of species to try and identify general trends, if any exist.

## IV. Organizing and modifying the colour, shape and texture of petal pavement cells

**Fig. 3:**
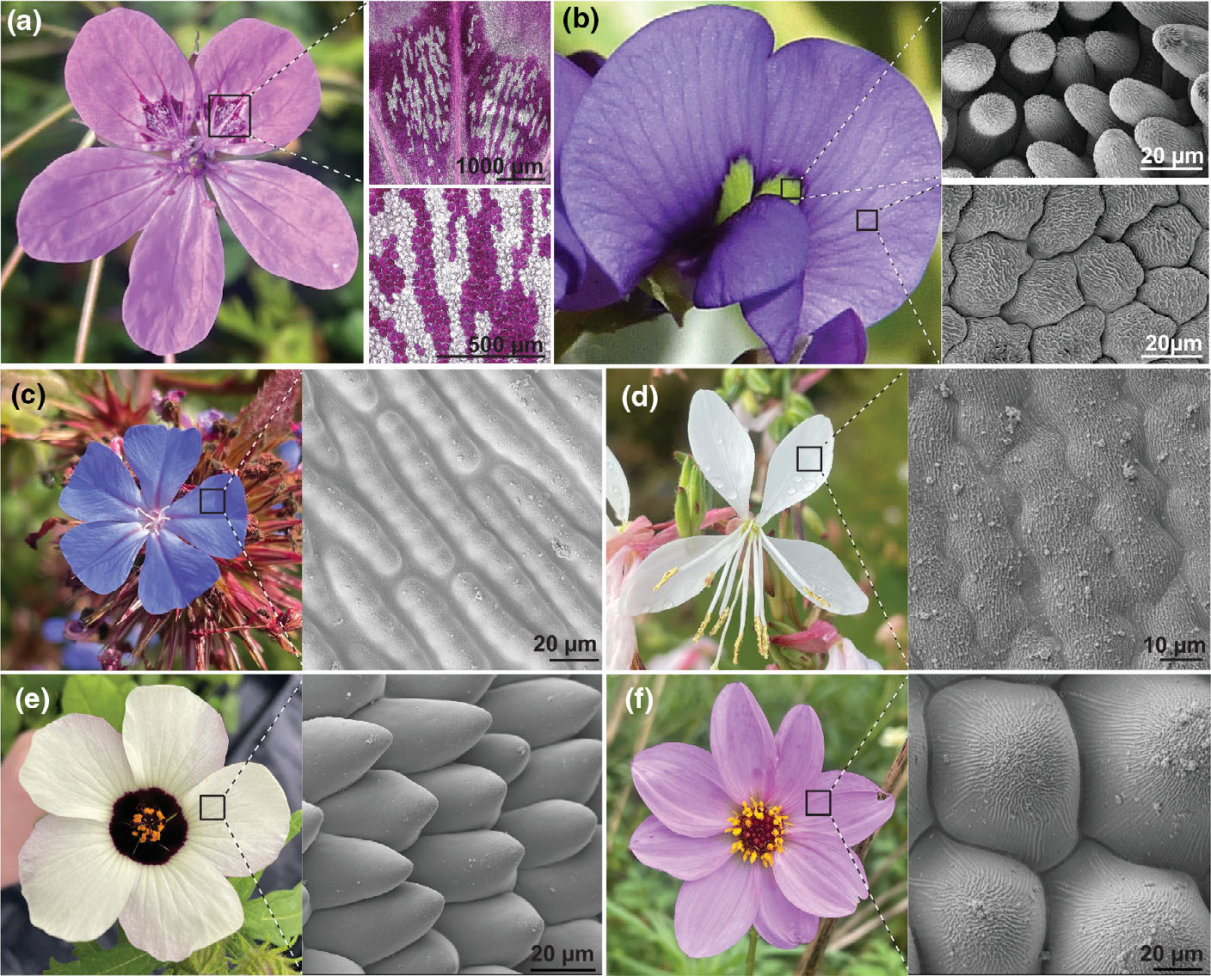
Micropatterns on the petal surface are created by variation in epidermal cell pigment, shape and cuticle textures. (a) Patterns on the petal of *Erodium castellanum* (Geraniaceae) created by cell‐specific pigmentation. (b) Variation in cell shape across the petals of *Hardenbergia violaceae* (Fabaceae). (c) Smooth cuticle on elongated petal epidermal cells of *Ceratostigma griffithii* (Plumbaginaceae). (d) Wrinkled cuticle on the elongated petal epidermal cells of *Gaura lindheimeri* (Onagraceae). (e) Smooth cuticle on the conical petal epidermal cells of *Hibiscus trionum* (Malvaceae). (f) Wrinkled cuticle on the conical petal epidermal cells of *Dahlia merckii* (Asteraceae).

Patterns emerge across the petal surface when pavement cells in distinct regions of the epidermis display contrasting characteristics: Petal epidermal cells can vary in shape, texture of their waxy cuticle and pigment content (Fig. 3). Recent studies have begun to identify the mechanisms involved in programming and modifying pavement cell properties across the corolla.

### 1. Spatial control of petal pigmentation

Pigmented patterning on the petal surface is most well studied of these epidermal cell characteristics. The main classes of compounds that colour the corolla are flavonoids (red to blue anthocyanins, yellow chalcones and aurones or UV‐absorbing flavonols), carotenoids (reds, orange and yellows), betalaïns (reds and blues, exclusive to the Caryophyllales) and chlorophylls (greens), and their biosynthesis pathways are well understood (Tanaka *et al*., 2008; Tripathy & Pattanayak, 2012). The chemical properties of these molecules are highly modifiable, such as the addition/removal of sugar or methyl moieties or changes in pH, which allows for exceptional variation in pigmentation across flowers (Morita *et al*., 2015; Stavenga *et al*., 2021). Colourful petal patterns are created by the precise production of these compounds in different epidermal cells across the petal surface (Fig. 3a; Fattorini *et al*., 2024; Lin & Rausher, 2021b; Yuan *et al*., 2023). Occasionally, pigments in underlying mesophyll cells can also contribute to the overall effect of pigmentation patterns (Cavallini‐Speisser *et al*., 2021; Yuan *et al*., 2023).

**Fig. 4:**
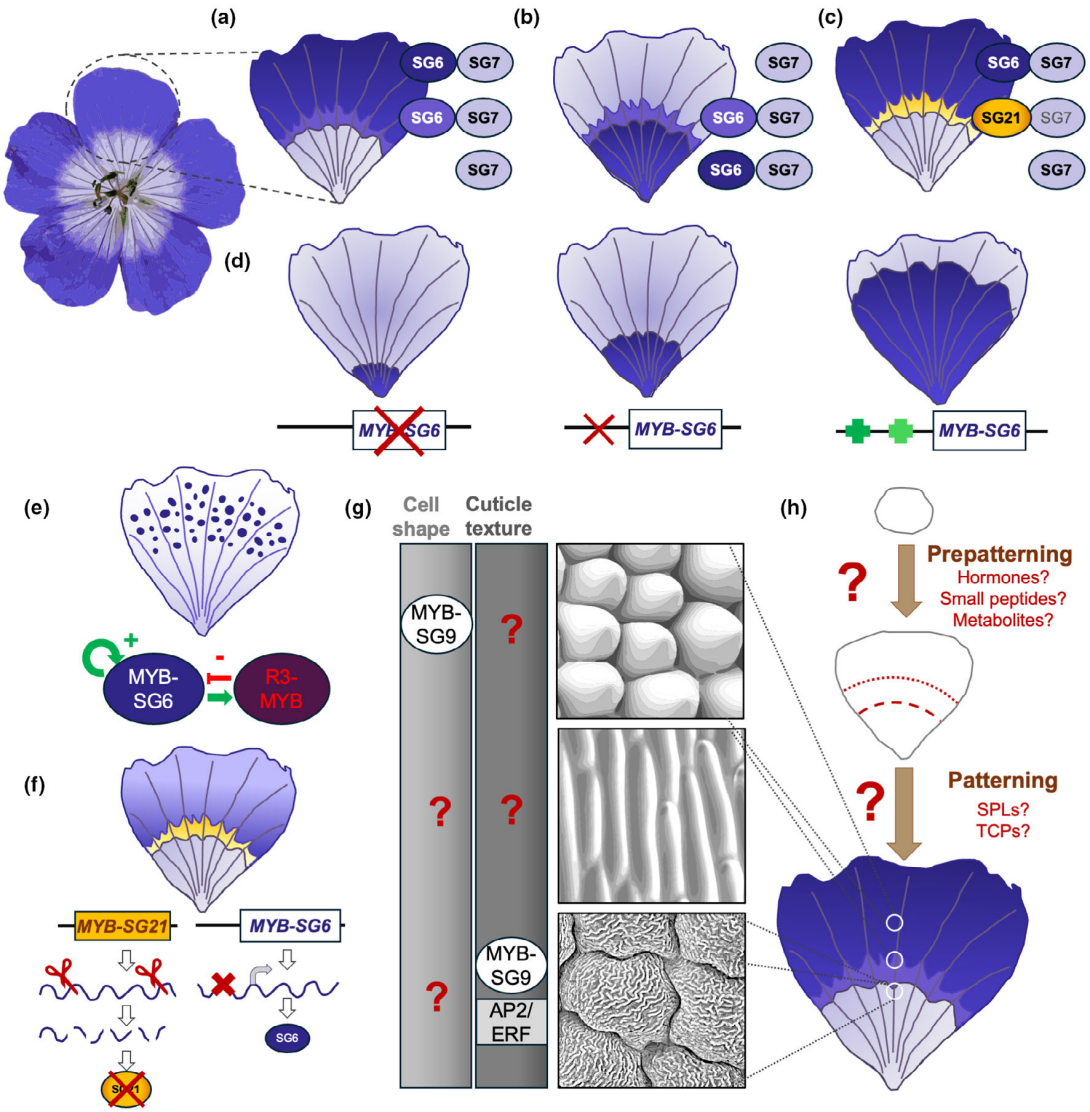
*MYB* transcription factors are major contributors to petal pattern development and evolution. (a) Spatial restriction of *MYB* gene expression (e.g. subgroup 6 (SG6) R2R3‐myeloblastosis (MYB) promote anthocyanin production while subgroup 7 (SG7) R2R3‐MYB activate flavonol synthesis) largely accounts for pigmentation pattern formation during petal development; (b) variation in expression domain or (c) in the identity of the *MYB* gene expressed in a given region (subgroup 21 R2R3‐MYB can promote the production of yellow carotenoids) can generate the intra‐ and interspecific diversity in petal patterns. (d) Variation in pattern proportions can be due to mutation affecting the coding sequence or the regulatory region of *MYB* genes. Red crosses depict deleterious mutations, and green crosses represent gain of *cis*‐regulatory elements. (e) Petal pigmentation spots can emerge from the interactions between a transcriptional activator (SG6 R2R3‐MYB) and a repressor (e.g. R3‐MYB). Green arrows indicate transcriptional activation, red blunt arrow indicate transcriptional repression. (f) Post‐transcriptional processes targeting SG6 R2R3‐MYBs can also yield petal pattern variation, reducing pigment production via changes in the 5′UTR impacting protein translation (right) or abolishing pigment production in certain petal domains via siRNA promoting the degradation of SG6 R2R3‐MYB transcripts (left). Arrows indicate transcription and translation steps. (g) *MYB* genes are also involved in specifying cell shape and cuticular texture, but many outstanding questions remain: Beyond bHLH/WD40 that help MYB regulate anthocyanin production and AP2/ERF factors that participate in cuticular ridges formation, most of the molecular players working along MYBs to control epidermis cell characteristics are yet to be identified. What specifies the geometry of nonconical cell shape and what decides whether SG9 MYBs induce conical cell or trichome formation is also not understood. (h) Crucially, the signalling events and upstream regulators that specify petal polarity, pattern cell fate along the axes of developing petal primordia and divide the petal surface into distinct territories where growth can be controlled independently, and neighbouring cells can acquire contrasting fates remain unclear. Brown arrows indicate the developmental progression from emerging primordia on the floral meristem to mature petals in open flowers.

Evolving intricate pigmentation patterns, such as scattered speckles, eye‐like concentric spots or juxtaposed horizontal stripes, requires the genetic machinery that produces different pigments to be spatiotemporally restricted during development. This process is often regulated by the myeloblastosis (MYB)‐domain family of transcription factors (Zhao *et al*., 2022). For example, petal anthocyanin biosynthesis is regulated by MYB transcription factors as part of a trimeric complex also involving a bHLH TF and a WD40 protein (MBW complex) that transcriptionally activates different pigment biosynthetic genes (Xu *et al*., 2015). MYB‐bHLH‐WD complexes also regulate carotenoid and betalaïn biosynthetic processes in many species, suggesting that these pathways are evolutionarily conserved (Hatlestad *et al*., 2015; Sagawa *et al*., 2016; Lloyd *et al*., 2017; Zhao *et al*., 2022). The spatial restriction of *MYB* genes to specific subdomains of the emerging petal primordia often accounts for the formation of pigmentation patterns (Fig. 4a–c). Variation in the expression pattern of MYBs and their co‐activators accounts for the majority of flower colour variation across and between species (Fig. 4d–f), as surveys of genetic variation in flower pigmentation across species found that the genes encoding the MBW complexes themselves display faster rates of molecular evolution than their downstream biosynthetic enzymes (Schwinn *et al*., 2006; Wessinger & Rausher, 2012; Wheeler *et al*., 2021, 2022). This phenomenon is likely adaptive, as it allows for the preservation of pigment biosynthesis pathways for other processes, such as stress responses and the pigmentation of other organs (Davies *et al*., 2018).

Diversification of pigmentation patterns due to evolutionary drivers are mediated by changes in the composition, type and relative position of the different elements of a pattern. From a theoretical viewpoint, petal pigmentation patterns can be formed and diversify following mechanisms similar to those responsible for colourful motifs on the skin or fur of animals (Galipot *et al*., 2021). Repetitive patterns like small, scattered spots can emerge via self‐organization following the Turing model and only require interactions between a transcriptional activator and a repressor (Turing, 1990; Meinhardt, 2012). In this model, expression of the inhibitor is promoted by the activator. The inhibitor then represses the pigmentation activator in nearby tissue, creating spots or stripes across an organ (Fig. 4e; Galipot *et al*., 2021).

The Turing reaction–diffusion model has been validated both *in silico* and *in vivo* in *Mimulus* (Fig. 1i). Red spots on the nectar guide of the yellow petals of *M. lewisii* are created by synchrony between *RTO*, a MYB repressor of anthocyanin production, and NEGAN, a subgroup 6 R2R3‐MYB that activates anthocyanin production. In red‐spotted regions, NEGAN promotes anthocyanin biosynthesis and *RTO* expression. An active RTO protein can move into adjacent tissues to repress *NEGAN* expression and anthocyanin production, maintaining yellow tissue around the red spot (Fig. 4e). This phenotype is likely also adaptive, as a loss‐of‐function mutation in *RTO* produces a ‘red‐tongue’ phenotype, in which the discrete spots are replaced with a large blotch of colour, resembling wild *Mimulus* populations across California (Ding *et al*., 2020).

Computational approaches to model petal development have been extremely useful in understanding the emergence of distinct corolla patterns (Rolland‐Lagan *et al*., 2003; Green *et al*., 2010; Sauret‐Güeto *et al*., 2013; Rebocho *et al*., 2017; Zhang *et al*., 2024). More recently, theoretical models have been developed specifically to better understand the processes that led to the emergence of various pigmentation patterns (Ringham *et al*., 2021; Simmons *et al*., 2023; Liang *et al*., 2025). Genes do not work in isolation but instead interact with each other, forming information processing webs, commonly known as gene regulatory networks (GRNs), that control cell behaviour, such as differentiation and growth. Novel *in silico* simulations of developing petals at cellular resolution should also allow us to investigate how the GRNs that govern petal patterning originate and how complex patterns evolve (Rolland‐Lagan *et al*., 2003; Ringham *et al*., 2021; Zhang *et al*., 2024). Early attempts are promising: They demonstrate how the spontaneous specification of an additional boundary cell type can contribute to the emergence of bullseye patterns, uncover the importance of noise in GRN to generate robust petal motifs and establish growth as a central player to maintain or modify pattern proportions as petals develop (Riglet *et al*., 2024; Oud *et al*., 2025).

Recent gene duplication events play a central role in the diversification of petal pigmentation patterns, allowing closely related MYBs to acquire distinct expression patterns and/or biochemical properties. This accounts for the production of the complex eyebrow‐like horizontal stripes of *Nigella orientalis* (Yuan *et al*., 2023) and the formation of red spots and white cups on the petals of *Clarkia gracilis* (Lin & Rausher, 2021b). Differences in the positioning of red petal spots and relative size of the white cup domain between subspecies of *C. gracilis* are also due to loss‐of‐function mutations, gene loss and *cis*‐regulatory changes affecting the same *MYB* paralogues (Fig. 4d; Lin & Rausher, 2021a).

MYBs can also regulate UV patterns. For example, sunflowers and many other *Asteraceae* have UV bullseye patterns created by the spatial restriction of UV‐absorbing flavonoids to the proximal region of the corolla in ray florets, the peripheral flowers of the Asteraceae inflorescence (Moyers *et al*., 2017; Fig. 2c–e). A study in wild sunflowers uncovered that intraspecific differences in UV‐absorbing bullseye are controlled by *cis*‐regulatory variation in a *MYB* gene (Fig. 4d; Todesco *et al*., 2022). This work, along with other recent investigations (Koski & Ashman, 2015; Bradley *et al*., 2017; Ding *et al*., 2020), underscores that natural variation is a powerful tool to investigate the genetic basis of petal pattern diversification. It also provides a path to identify the upstream processes regulating *MYB* expression, as polymorphisms in the promoter region of *HaMYB111* that associate with bullseye dimensions could help identify the binding sites of upstream transcription factors.

While changes to gene transcription have been the dogma for the evolution of petal pigmentation, new studies indicate that the regulation of complex patterns also rely on posttranscriptional mechanisms (Fig. 4e). In *Mimulus lewisii*, the *YELLOW UPPER* (*YUP*) locus produces siRNAs that silence *REDUCED CAROTENOID PIGMENTATION2* (*RCP2*), a *MYB* regulator of carotenoid production (Liang *et al*., 2023). Variation in the *YUP* locus accounts for the production of a yellow nectar guide on the pink corolla of the bee‐pollinated *M. lewisii* or the uniform red pigmentation of the bird‐pollinated *M. cardinalis* and swapping *YUP* alleles between species is sufficient to modify pollinator preference (Bradshaw & Schemske, 2003). Together, this suggests that this small RNA locus played a key role in diversification and pollinator‐mediated reproductive isolation in closely related *Mimulus* species (Peng *et al*., 2024).

These findings complement earlier research in wild populations of snapdragon (*Antirrhinum majus*) subspecies *Antirrhinum majus subsp. striatum* and *Antirrhinum majus subsp. pseudomajus* (Whibley *et al*., 2006). Researchers found a small RNA named *SULF* that targets chalcone 4′‐O‐glucosyltransferase (*Am4′CGT*), a gene that is important in synthesis of the yellow pigment (Bradley *et al*., 2017). *Am4′CGT* and *SULF* expression patterns are complementary, suggesting that SULF posttranscriptionally represses *Am4′CGT*. SULF is also epistatic to a MYB‐domain transcription factor regulating red pigment synthesis, *ROSEA* (*ROS*), which highlights complex mechanisms for regulating different pigments across the petal (Whibley *et al*., 2006; Bradley *et al*., 2017). In addition, *SULF* is under selection in naturally hybridizing populations of *A. majus subsp. striatum* and *A. majus subsp. pseudomajus*, indicating this important in the evolution of petal patterning in *Antirrhinum*.

Interestingly, both of *SULF* and *YUP* are taxa‐specific and were a result of by incomplete or inverted duplication of protein‐coding genes. In *Mimulus*, the partially copied gene is a CYP450 homologue that is unrelated to pigment production (Liang *et al*., 2023). Together, these studies highlight the role of posttranscriptional processes in the evolution of complex pigmentation patterns – it should also encourage researchers mapping quantitative trait loci in other model systems to look beyond protein‐coding genes.

### 2. Patterning cell shape across the petal epidermis

Epidermal cells can also vary in size, shape and texture across the petal (Fig. 3b–f; Christensen & Hansen, 1998; Kay *et al*., 1981). The distribution of these structural characteristics is controlled with remarkable precision: The petal surface of *Nigella arvensis* involves the arrangement of more than eight different cell shapes (Yao *et al*., 2019). Petal cell shape can also vary extensively between species and different petals of zygomorphic flowers often exhibit distinct cell geometries patterns (Bailes *et al*., 2018; Liao *et al*., 2020).

Variation in cell shape across the petal likely has an adaptive role and can impact pollinator preference. For example, in isolated populations on the Macaronesian islands, the loss of conical cell shape on the petal epidermis has occurred independently at least five times in eudicots species that have shifted from primarily insect to bird pollination (Ojeda *et al*., 2016). However, a more recent comparative study of species‐pairs across 13 genera (11 asterids and 2 rosids) found no correlation between petal epidermal cell shape or texture and pollination mechanism (Kraaij & van der Kooi, 2020). This suggests the ecological relevance of petal epidermal cell shape variation may differ between species and emphasizes the need for more detailed characterization of the contribution of cell shape to angiosperm evolution.

The most well‐studied petal epidermal cell shape is a dome‐like cone (Fig. 3e,f). These conical cells are present on the petals of most species, often found combined with other cell shapes (Kay *et al*., 1981). Conical cells contribute to pollinator attraction by enhancing the colour of the flower (Noda *et al*., 1994) and are also important for physical properties of the petal, including preventing petal wettability, regulating flower temperature and providing grip for pollinating insects (Whitney *et al*., 2009). Subgroup 9 MYB transcription factors, also known as *MIXTA* and *MIXTA‐like* genes, regulate conical cell formation (Fig. 4g; Brockington *et al*., 2013; Reed *et al*., 2022). *MIXTA‐like* genes are widely conserved as regulators of epidermal cell shape across angiosperms and variation in conical cell distribution across the petal correlates with their expression pattern during development. For example in *Thalictrum*, the loss of conical cell shape corresponds with changes in expression of a *MIXTA‐like* gene and is correlated with the transition from insect to wind pollination (Di Stilio *et al*., 2009). However, the identity of the transcriptional regulators specifying other petal epidermal cell shapes is still mysterious (Fig. 4g). Overall, the GRNs controlling how and where distinct cellular geometries emerge in a robust manner across the corolla are still largely unknown and are important future research venues (Cavallini‐Speisser *et al*., 2021; Riglet *et al*., 2021).

### 3. Cell texture: Variation in cuticle properties across the petal epidermis

A third, less appreciated element of petal epidermal patterning is the cuticle texture. A wide range of cuticular patterns exists across the petals of angiosperms, and research over the last few years has started to highlight their contribution to corolla diversification (Fig. 3c–f). The cuticle texture can vary across a single petal (Moyroud *et al*., 2022) and contributes to the corolla appearance: Disordered striations created by alternate ridges and grooves on the cuticle of petal epidermal cells contribute to structural colour and UV patterns yielding visual cues to pollinators (Moyroud *et al*., 2017). Cuticular patterns also act as water repellent, influence temperature on the corolla surface and impact attachment and flower handling by visitors (Koch *et al*., 2009; Bräuer *et al*., 2017; Wilts *et al*., 2018; Gorb & Gorb, 2023). Cuticle structure likely influences floral scent emission and produces the ‘velvety’ and ‘waxy’ textures of orchid petals that prevent insect adhesion (Prüm *et al*., 2011; Xiao *et al*., 2020; Liao *et al*., 2021).

Theoretical models proposed that the rate of cuticle production, the extent and direction of cell expansion, the relative stiffness of the cuticle and the underlying cell wall properties are all important to control the production, direction and regularity of ridges on petal epidermal cells (Antoniou Kourounioti *et al*., 2013; Airoldi *et al*., 2021; Lugo *et al*., 2023). The chemical composition of the cuticle also influences its material properties and varies between species (Kunst & Samuels, 2009). For example, the presence of cuticular striations in flowers belonging to a group of *Hibiscus* species native to Australia and New‐Zealand (Fig. 2f) is best predicted by examining the accumulation of waxes and phenolic compounds in the petal epidermis cuticle (Moyroud *et al*., 2022).

*SHINE* transcription factors from the *AP2/ERF* family were first identified as regulators of cuticle ridge formation in *A. thaliana* petals (Fig. 4g; Shi *et al*., 2011; Oshima *et al*., 2013). Work done in *Hibiscus trionum* suggests that gene duplication followed by functional divergence could be central to the emergence of different cuticular patterns across the petal. *H. trionum* petals exhibit flat striated cells in the proximal portion and smooth conical cells in the top region, with different cuticle chemical profiles between the two domains (Fig. 2f; Giorio *et al*., 2015). While *HtSHINE1* and *HtSHINE2* are lowly expressed across the emerging petal, *HtSHINE3* is highly transcribed only in the striated domain and can modify cuticle composition and induce ectopic striations when constitutively overexpressed (Moyroud *et al*., 2022).

*MYB*‐domain transcription factors also participate in the control of cuticle production and ridges formation (Fig. 4g; Xu *et al*., 2021). In *A. thaliana*, *MIXTA*‐like transcription factors, together with *SHINE* genes, act redundantly to induce conical cell formation and striation emergence (Oshima *et al*., 2013; Shi *et al*., 2011), providing a simple way to co‐regulate both cell shape and texture. However, this is not conserved across all species, as *HtMIXTA‐like1* regulates cuticle composition and striation production but does not participate in the specification of the conical cell shape on *H. trionum* petals (Moyroud *et al*., 2022).

### 4. Petal pavement cell features work in combination

Although a systematic investigation across the angiosperms is lacking, targeted studies indicate that changes in epidermal cellular features often occur in combination with each other, creating petal subdomains with distinct properties (Berry & Geeta, 2019; Riglet *et al*., 2021; Berry *et al*., 2023). For example, floral UV patterns can be created through variation in cell pigments, shapes and cuticle texture, or a combination of the three (Fig. 2a–f). UV‐absorbing pigments (flavonoids, carotenoids but also other compounds like lignin) have long been known to produce motifs on the corolla. However, more recent studies revealed that structural characteristics of epidermal cells also matter: cuticular striations, when associated with flat elongated cells, can act as semi‐ordered diffraction gratings, producing blue/UV‐reflective cues that pollinators can perceive (Moyroud *et al*., 2017). The cell architecture of the petal surface can also enhance UV absorption on its own, as replicas of corolla surface using transparent resin still produce UV patterns mimicking those present on the surface of real flowers (Fig. 2f; Schulte *et al*., 2019).

Petal cell pigmentation, shape and texture can work together to mediate evolutionary innovations. The Beetle daisy (*Gorteria diffusa*) exhibits a remarkable range of intraspecific variation, with different floral morphs found in geographically distinct populations (Ellis & Johnson, 2009; Thomas *et al*., 2009). Some populations display raised black spots on petal‐like ray florets that resemble the primary pollinator of this species, a small bee‐fly called *Megapalpus nitidus*. These spots are a form of visual mimicry, as they resemble female bee‐flies that attract males pollinators through sexual deception (Johnson & Midgley, 1997). Such elaborate tridimensional structures are created by localized changes in epidermal cell pigment, shape, texture and UV reflectance, each contributing to the complex appearance of petal spots (Johnson & Midgley, 1997; Thomas *et al*., 2009; Fattorini *et al*., 2024).

Surprisingly, the genetic pathways associated with petal spots in Beetle daisy participate in iron homeostasis, root hair production and control of the juvenile to adult vegetative transition (Kellenberger *et al*., 2023). These gene networks contribute to unrelated processes may have been sequentially co‐opted to facilitate the creation of a phenotypic novelty and take advantage of insect mating behaviour to favour pollination. For example, the miR156/SPL module is known to be important early in vegetative development, but a role in specifying petal spots, where miR156 was not known to be highly expressed, is an exciting novel function for the evolution of this pathway (Poethig & Fouracre, 2024). While the molecular mechanism by which these developmental pathways are repurposed to create these spots is not yet understood, it provides an interesting example of how evolution can drastically alter and combine different epidermal cell traits to create diversity on the petal surface. The development of protocols to genetically manipulate Beetle daisy will be a promising venue to test the hypothesis that combined gene co‐options enabled the rapid evolution of a novel and complex trait. It would also be interesting to know whether other unrelated South African species of *Gazania, Dimorphotheca* and *Pelargonium* that display similar dark spots (Johnson & Midgley, 1997) evolved these patterns using the same genetic pathways.

### 5. Beyond MYBs: revealing the upstream mechanisms patterning the petal epidermis

Little is known about the upstream regulators of *MYBs* associated with petal pigmentation and the nature of their contribution, if any, to the diversification of colour patterns (Fig. 4h). Recent research has uncovered some candidate transcription factors which could regulate the expression of the MBW complex: In the Beetle daisy (*Gorteria diffusa*), presence of a pigmented black spot on petals coincides with high expression of a *GdSPL1*, a *SQUAMOSA PROMOTER BINDING PROTEIN‐LIKE* transcription factor. Interestingly *GdSPL1* is silenced in unspotted petals by the microRNA, miR156 and co‐option of the miR156‐*SPL* module (a gene network involved in juvenile to adult leaf development) could have been instrumental in establishing spot placement (Kellenberger *et al*., 2023). However, whether Gd*SPL1* promotes anthocyanin production and acts as an upstream regulator of the MBW complex to create the darkly pigmented spot characteristic of this species remains to be tested. Similarly, little is known about the upstream processes responsible for the spatiotemporal control of *MIXTA* and *MIXTA‐like* genes expression to restrict conical cell production or the formation of cuticular striations to certain petal domains (Fig. 4g). In *Lotus japonicus*, a *CYCLOIDEA* homologue, *LjCYC2*, activates the expression of subgroup 9 MYBs to restrict conical cell production to the very base of wing petals (Ojeda *et al*., 2017). Another *CYC* homologue, TfCYC2 in *Torenia fournieri*, was shown to directly bind to the promoter of *TfMYB1*, a regulator of pigment production in this species, to promote its expression during petal development (Su *et al*., 2017). However, whether *CYC* genes act upstream of *MYBs* to regulate cell shape or pigmentation in other species has not been examined.

More generally, the upstream regulatory events that restrict the expression of *MYBs* and other transcription factors that control pigment synthesis, cell shape or cuticular texture to specific domains of the corolla remain largely obscure, but new research suggests these patterns are defined very early in development (Fig. 4h). Recent data in *Hibiscus* indicate that the petal epidermis is divided into distinct subdomains when developmental boundaries are set long before any visible pattern emerges on its surface (Riglet *et al*., 2024). This creates a ‘paint‐by‐number’ canvas, allowing cell behaviour to be controlled independently in different subregions. Importantly, variation in the positioning of the prepattern boundary coincides with a change in bullseye dimension between *H. trionum* and its sister‐species *H. richardsonii*. Changes in pattern proportions could have contributed to speciation in these two species, as buff‐tailed bumblebees exhibit a strong preference for the larger bullseye of *H. trionum* over the smaller pattern of *H. richardsonii* (Riglet *et al*., 2024). However, the molecular players, signalling processes and cellular events involved in partitioning the corolla so early in development are still unknown. Signalling events that specify petal polarity are likely to play a key role in patterning cell fate specification and growth across the surface of developing petal primordia (Cavallini‐Speisser *et al*., 2021). Hormones, like auxin, are obvious candidates (Salvi & Moyroud, 2025), but other molecular players like small peptides or secondary metabolites should also be considered (Fig. 4h).

High spatial resolution approaches, such as single‐cell or single‐nuclei RNA‐seq, should greatly enhance our understanding of prepatterning events and cell fate specification across the corolla. These technologies are rapidly evolving and have yet to be applied to many tissue types and developmental trajectories (Zhang *et al*., 2021; Shahan *et al*., 2022; Zhu *et al*., 2022). However, their use in recent petal studies led to interesting findings clarifying the spatiotemporal dynamics of petal volatile compounds (Kang *et al*., 2022; Guo *et al*., 2024) and metabolite production (Neumann *et al*., 2022). While spatial transcriptomic methods are still in their infancy in plants (Rodriguez‐Villalon & Brady, 2019; Zhu *et al*., 2022), together with single‐cell metabolomic approaches (Misra *et al*., 2014; Fujii *et al*., 2015; Samarah *et al*., 2020), they provide promising avenues for understanding how micropatterns emerge across the corolla at single‐cell resolution.

## V. Diversifying petals by changing cell type specification: The case of floral trichomes

**Fig. 5:**
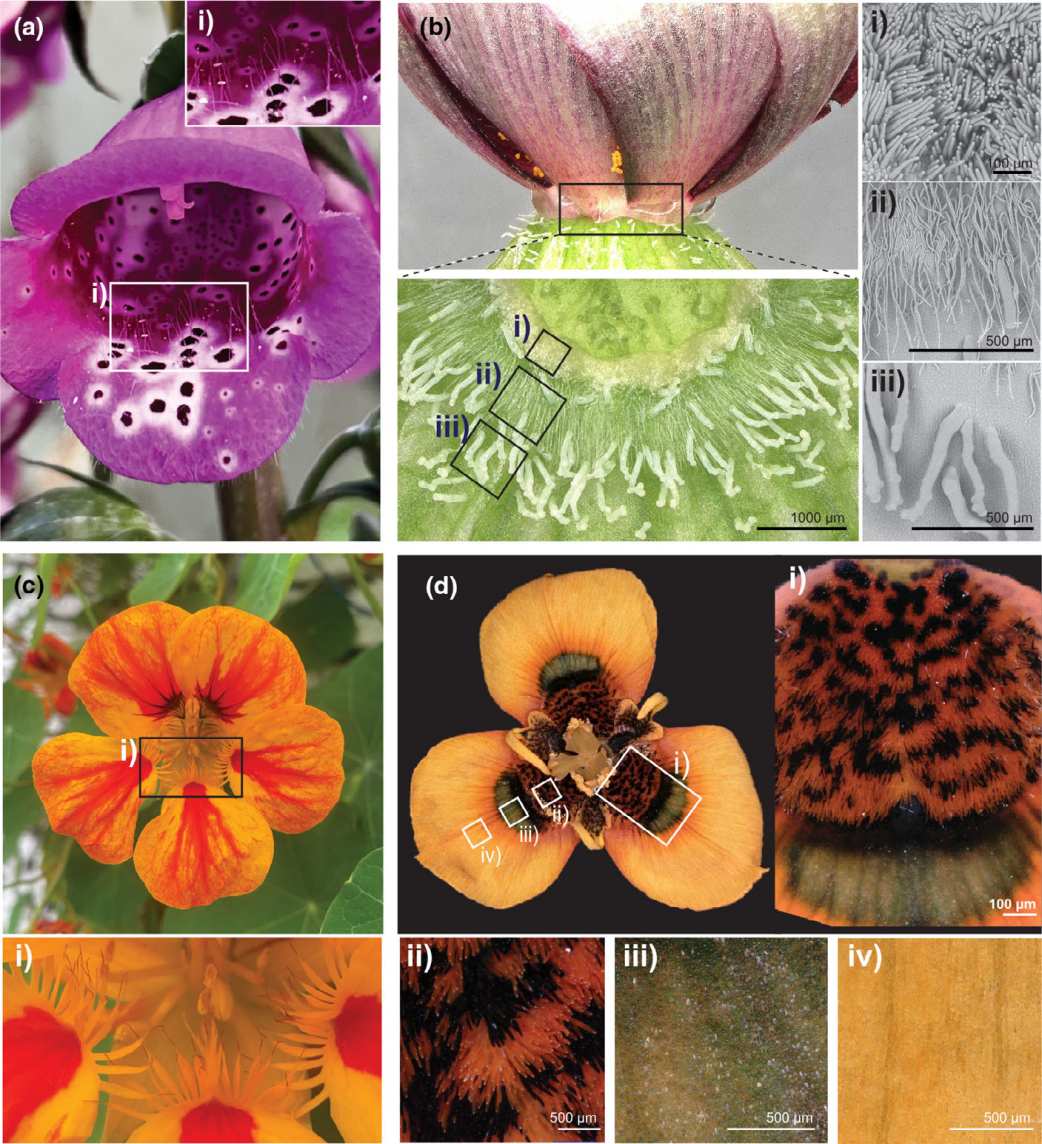
Variation in trichome‐like structures creates floral micropatterns. (a) *Digitalis purpurea* (Foxglove) with elongated trichomes on its corolla (i). (b) Three trichome domains in the floral nectary of *Hibiscus trionum*. (i) Nectar‐secreting trichomes closest to the base of the flower, (ii) single‐celled defensive trichomes in the middle region and (iii) enlarged glandular trichomes at the periphery of the nectary. (c) The petal of *Tropaeolum majus* (Nasturtium) with (i) serration‐like outgrowths. (d) The petal of *Moraea tulbaghensis* with different epidermal cell types in three discrete domains (i). (ii) Cilia‐like structures in the proximal region, (iii) iridescent conical cells in the middle region and (iv) elongated yellow cells at the distal region of the petal.

Although pavement cells constitute most of the petal epidermis, the corolla of many species also exhibits a remarkable diversity of trichomes (Fig. 5a,b). These plant hairs can be made of one or several cells, be glandular or nonsecretory and constitute highly specialized structures – their morphology, metabolic capabilities and distribution influence their functionality (Werker, 2000). Petal trichomes contribute to many aspects of pollination: They produce scents that attract pollinators (Kolosova *et al*., 2001; Marinho *et al*., 2014; Meinhardt, 2012; Ghissing & Mitra, 2022), form optical cues (Lam *et al*., 1980) and provide postlanding guides helping pollinator access pollen and nectar (Chen & Yuan, 2024). Glandular trichomes can also synthesize nectar or constitute edible rewards themselves when they contain starch grains, oil droplets or protein bodies (Pansarin & Maciel, 2017). Trichome identity and distribution across petals vary both within and between species (Muravnik *et al*., 2022). For example, trichome type differs between ray and disc florets of daisies (Thomas *et al*., 2009) and between the different petals forming the zygomorphic corolla of legume flowers (Ojeda *et al*., 2009). Thus, the precise specification of epidermal cell fate (pavement cell vs trichome) is a key component in patterning the corolla surface at the microscale.

Compared with leaves, the mechanisms specifying trichome identity and distribution on petals are not well understood. Investigations in a few species identified HD‐ZIP and MYB transcription factors as key regulators (Wu *et al*., 2023; Zahid *et al*., 2023), but it is not clear whether distinct trichome types are regulated by distinct *MYBs* or whether a single *MYB* can produce different trichomes types when the timing or level of its expression, or the availability of its co‐regulators vary. Studies on petal trichomes suggest that the regulators involved differ from that of other organs: While subgroup 9 MYBs (*MIXTA‐like*) regulate hair fate on the corolla of several species (e.g. Tan *et al*., 2016; Chen & Yuan, 2024), leaf trichome development is controlled by MYB subgroup 15 members, at least in Arabidopsis. Specialization within subgroup 9 has occurred to regulate petal (*GhMYB10*) and seed trichomes (*GhMYB25* and *GhMYB25‐like*) in cotton, suggesting that gene duplication and functionalization could be instrumental in the diversification of trichome type. Reciprocally, the tufted trichomes (unicellular elongated hairs) at the base of the cotton petal are regulated by *GhMYB25* and *GhMYB25‐like*, and subsequent co‐option of the associated regulatory network in the seed epidermis could be a first step towards the evolution of cotton fibres (Tan *et al*., 2020).

Assemblages of petal trichomes form nectar guides in *Mimulus*, but what controls interspecific variation in this trait was not understood. Recent findings start to fill the gap, showing that *GUIDELESS*, a subgroup 9 *MIXTA‐like MYB*, underpins differences in trichome length between the nectar guides of two *Mimulus* species (Chen & Yuan, 2024). In the bumblebee‐pollinated *M. lewisii, GUIDELESS* promotes the production of long trichomes along the nectar guides. In the self‐pollinated *M. parishii*, a deleterious SNP in *GUIDELESS* yields shorter trichomes. However, the mechanism enabling GUIDELESS to regulate trichome length is still not understood. In addition, *GUIDELESS* is not the only locus regulating nectar guide trichome length between these species, suggesting that other unknown genetic factors have contributed to a switch in mating strategy from bumblebee‐mediated pollination to selfing.

Hook‐shaped trichomes on the surface of the petal‐like sepals of *Aristolochia esperanzae* are central to pollen removal and fruit set: shaved flowers capture < 1% of the pollinators caught by a trichome‐bearing flower and fail to produce fruits (Matallana‐Puerto *et al*., 2024). Trichomes distribution, orientation and morphology directly contribute to the efficiency of the trap. However, a closely related species, *A. macrophylla*, lacks such pollen‐trapping trichomes, suggesting that there could be other evolutionarily significance to their presence or absence (Suárez‐Baron *et al*., 2023). Elucidating the mechanisms governing patterning of these trichomes should contribute to our understanding of a structure that directly affects reproductive fitness: transcriptomic studies identified various MYBs (*AfimGL2, AfimMYB106*‐like) and members of the AP2/EREBP family (*AfimRAV1*‐like and *AfimWIN1*) as candidate to control trichome development on the perianth of *A. fimbriata* flowers (Suárez‐Baron *et al*., 2021). However, further work is necessary to functionally validate those genes and determine whether they could also regulate trichomes specification in petals and petaloid sepals of other species.

Finally, certain trichomes specialize in surprising ways: In cotton, trichomes on the outer epidermis of the corolla act as glue that holds adjacent petals together. These trichomes fulfil a mechanical role, ensuring correct bud shape and protecting reproductive organs from desiccation during development (Tan *et al*., 2016). The force required to separate petals from each other quantitatively relates to the extent of entanglement between stellate trichomes on adjacent petals. Correct patterning of cell fate is essential to achieve this ‘velcro effect’ as trichomes must reside on the part of the abaxial epidermis in direct contact with neighbouring petals, but here too, mechanisms controlling patterning are unknown.

Due to their diversity in both type and location, petal hairs provide a great opportunity to start exploring the role physical forces play in the evolution of flower form – an area currently understudied.

## VI. Patterning cell behaviour supports the evolution of novel petal‐associated features

Petals in many species have highly elaborate shapes and appendages (Fig. 5c,d) that can be extraordinarily diverse (Endress & Matthews, 2006). These structures are excellent systems to explore general patterns of flowering plant diversification and understand evolution innovates, yet how they emerge is mostly unknown. How morphological novelties originate is an important question in evolutionary biology (Wagner, 2015), as evolutionary innovation led to novel functional capacities or new body parts. Controlling cell behaviour spatially across the corolla is essential for both and below we consider two specific cases.

Nectar producing glands, or nectaries, have a direct impact on pollinator attraction and contribute to speciation and evolution (Katzer *et al*., 2019; Parachnowitsch *et al*., 2019). Nectaries have evolved numerous times independently across angiosperms and are extremely diverse in respect to their location, structures and secretion mechanisms (Heil, 2011; Liao *et al*., 2025). The production of nectar relies on the development of specialized cell types, allowing us to explore evolutionary innovation at the cellular scale. In some species, trichome assemblage can form the basis of nectaries. For example, the nectaries of *H. trionum* and other Malvaceae are made of two concentric rings of hairs adjacent to the abaxial petal surface (Fig. 5d; Hu *et al*., 2020). Glandular trichomes closest to the petal base produce nectar while the outer whorl of uniseriate trichomes might play a defensive role (Fig. 5d). Elucidating the processes mediating the specification of two distinct hair types next to each other to generate a multifunctional structure represents a promising venue to understand how evolution generates a novel capacity (i.e. the ability to produce nectar). It will also be interesting to determine whether the mechanisms that mediate variation in nectary size and nectar volume produced by a flower is associated with pollination syndrome (Katzer *et al*., 2019).

In some species, nectar is produced at the bottom of long tubular outgrowths that restrict its access to visitors with a tongue long enough to reach it. These structures, called nectar spurs, can be derived from multiple floral organs (petals and sepals) and have evolved multiple times independently across diverse species of angiosperms (Hodges, 1997; Li *et al*., 2024). Morphological variation in the petal‐derived nectar spurs in *Aquilegia* can impact pollination: Long nectar spurs in North American populations of *Aquilegia* are associated with a reduction in pigment and gain of hawkmoths pollinators (Whittall & Hodges, 2007). In Asia, *Aquilegia* have evolved shorter nectar spurs while shifting to primarily bumblebee and scarid fly pollinators (Tang *et al*., 2007). Switching to the specialist hawkmoth as primary pollinator promotes outcrossing over the tendency for self‐pollination by more generalist bumblebees, suggesting that trade‐offs for the gain or loss of spurs impact reproductive isolation and speciation (Brunet & Sweet, 2006).

**Fig. 6:**
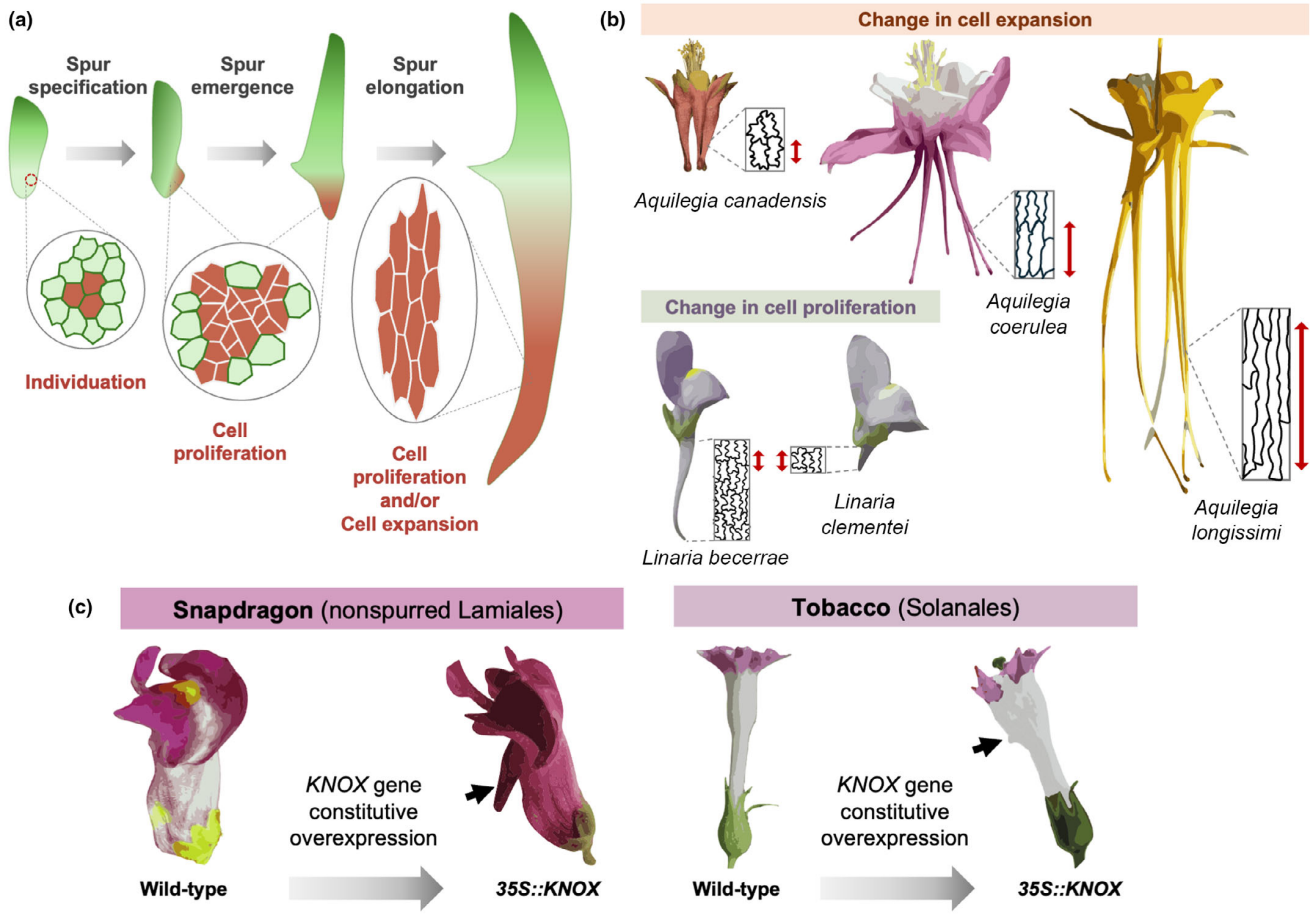
Individuation and change in growth at the cellular scale can support the evolution of novelty and biodiversity. (a) Nectar spur specification allows a subdomain of the petal primordia to become differentiated (individuation) and a spur emerges as cell proliferation is promoted in this ‘spur domain’. Depending on the genus, spur elongation is mostly driven through cell proliferation or cell expansion. (b) Interspecific variation in spur length is due to changes in cell expansion (differences in cell length/anisotropy) in *Aquilegia* (Puzey *et al*., 2012) and variation in cell proliferation (no difference in cell length but difference in number of cells) in *Linaria* (Cullen *et al*., 2018). Red arrows depict the average cell length in each case (c) Spur formation (black arrow) can be induced in spurless species, such as snapdragon (another species of Lamiales) or tobacco (Solanales), when a KNOX transcription factor promoting cell growth is constitutively overexpressed (Box *et al*., 2011).

Nectar spurs form when a group of cells in the emerging organ primordia experience enhanced cell division compared with the surrounding tissues (Fig. 6a). Subsequent spur elongation is driven through cell expansion or cell proliferation and different species use distinct strategies, and different genes, to achieve the final spur size. Cell length and anisotropy correlate with spur length in the Aquilegia genus (Puzey *et al*., 2012), but in *Linaria* species, cell size appears similar between species, and variation in spur dimension is due to differences in cell number (Cullen *et al*., 2018; Fig. 6b). This indicates that either process can be modulated to control spur dimensions. Remarkably, constitutive overexpression of a *KNOX* transcription factor associated with spur growth in snapdragon and tobacco, two spurless species, is sufficient to produce spur‐like appendages (Fig. 6c; Box *et al*., 2011), indicating that while these species lack the ability to grow spurs, they possess the programme allowing a group of cells to ‘break free’ from the rest of the petal. This suggests petal spur origination required two key events: the ability to specify the ‘spur spot’, to allow a group of cells to behave independently (individuation), followed by the independent recruitment of diverse ‘growth modules’ (genes controlling cell proliferation and/or expansion in the spur spot) along distinct lineages (Fig. 6a).

While KNOX genes promote cell proliferation and spur elongation in Lamiales, other species use distinct regulators to control spur growth. Mapping spur length in *Aquilegia* found that *POPOVICH* (*POP*), a zinc finger domain transcription factor, promotes cell proliferation during the early stages of spur development and loss‐of‐function mutations in *POP* accounts for loss of spurs in *Aquilegia ecalcarata*, suggesting that this locus is also an important contributor to petal spur evolution (Min *et al*., 2019; Ballerini *et al*., 2020). Spur elaboration in *Aquilegia* also depends on local repression of cell division by *TEOSINTE‐BRANCHED*‐like transcription factors (*TCP4*) and cell elongation likely driven by complex hormone signalling, including cytokinin, auxin, gibberellin and brassinosteroids (Yant *et al*., 2015; Conway *et al*., 2021). These pathways could also be involved in the evolution of nectar spurs in other species, as *TCP‐like* genes and hormonal responses have been proposed to regulate nectar spur length in *Linaria* and *Antirrhinum*, and the sepal/petal‐derived spurs of *Tropaeolum* (Cullen *et al*., 2023; Martínez‐Salazar *et al*., 2023).

However, the nature and origination of the signalling events that specify the spur spot in the first place are still poorly understood. To which extent those individuation processes merge with the prepatterning events that divide the petal surface allowing neighbouring cells to acquire distinct fates (see Section III and Fig. 4g) remains to be investigated. Resolving the mechanisms that control cell behaviour (growth and differentiation) across the petal with such spatial precision to produce autonomous subdomains would not only allow us to understand spur initiation but also shed light on events that enable evolution to innovate and create new body parts.

## VII. Drivers of natural selection on petal patterns and their contribution to speciation

Because change in patterns across the petal surface directly impacts plant reproductive success, variation in the processes that govern cell behaviour (growth and differentiation) and the emergence of these patterns constitutes a useful system for understanding the evolution of complex traits. However, there are limited studies that examine the level of selection on these traits across the genome. Here, we summarize what is known about the evolution of petal pigmentation, and how it can help us understand inter‐ and intraspecific adaptation and evolution.

### 1. Evolution of novel genetic material and selection of petal traits

For selection to act upon phenotypic variation, genetic variation must first emerge. Gene duplication and neofunctionalization are two main drivers of genome evolution (Clark, 2023). Gene duplication allows for the creation of new genetic material and yield paralogs that can evolve new functions. Indeed several transcription factor families have undergone extensive gene duplication and sequence divergence across the angiosperms (Irish, 2003; Zhang *et al*., 2023). Its impact on the diversification of petal patterns has been discussed throughout this review, mostly through cases from the *MYB* transcription factor family. Transposons are also a driver of evolution through the creation of genetic diversity across eukaryotes, and unsurprisingly, they also account for variation in petal pigmentation. Gene disruptions via transposons account for anthocyanin modification in *Petunia* (Nakajima *et al*., 2005) and rose (Li *et al*., 2022), white flower pigmentation in Chinese kale (Zhang *et al*., 2019), and variegated pigmentation patterns in varieties of Japanese morning glory (Iida *et al*., 2004).

Genome structure can facilitate reproductive isolation of closely related species by linking several genes that control species‐specific traits, insuring morphological characteristics segregate together (Gutiérrez‐Valencia *et al*., 2021). These speciation ‘super loci’ have been found in *Petunia*, *Mimulus* and *Anthurium*, and contain several genes that regulate key differences between species. For example, in *Petunia*, multiple traits associated with flower patterning and pollinator syndrome, including UV absorption, scent production and pistil length, are tightly linked, insuring they are inherited together (Hermann *et al*., 2013; Amrad *et al*., 2016). More recently, this locus was found to contain a gene that causes hybrid necrosis in plants that carry incompatible alleles, causing hybrids between *Petunia* species to be stunted and further promoting their separation in nature (Li *et al*., 2023). A similar phenomenon was observed in *Mimulus*. The *YUP* siRNA locus in *M. parishii* and *M. cardinalis* mentioned above is linked to two other *MYB* transcription factors genes *SISTER OF LIGHT AREAS1* (*SOLAR*) and *PETAL LOBE ANTHOCYANIN* (*PELAN*), both of which control aspects of petal pigmentation that influence pollinator preferences (Liang *et al*., 2023). Such ‘super loci’ provide an interesting mechanism for how closely related species can prevent cross pollination and hybridization in nature. Together, these findings help us understand how petal pigmentation patterns can contribute to reproductive isolation in natural populations and act as drivers of speciation events.

### 2. Evolution *in situ*: the importance of examining petal patterns in natural populations

To date, little has been done to understand how the genes regulating petal patterning behave under selection in natural populations. However, a handful of field studies underscore the contribution of patterned cell behaviour to evolution and speciation. For example, the genetic loci and networks regulating pigmentation in *Antirrhinum majus* are well understood. This basic knowledge has facilitated field studies to examine gene flow and natural selection in wild populations (Field *et al*., 2025). A recent study surveyed a natural hybrid zone that contains two subspecies of *A. majus* with distinct petal patterns. Pooled whole‐genome sequencing revealed that selective sweeps on two tightly linked *MYB* transcriptions factors that regulate pigment, *EL* and *ROS*, have prevented gene flow between distinct populations. These loci allow for discrete petal pigment phenotypes to be maintained and provide a potential mechanism for reproductive isolation and speciation in these populations (Tavares *et al*., 2018).

Examining nectar spur evolution in *Aquilegia coerulea* has revealed that gain or loss of nectar spurs can happen in a short time frame because of trade‐offs between pollinator preference and herbivory. In *Aquilegia*, the presence of long nectar spurs is an adaptation to pollination by hawkmoths, as their probiscis can reach nectar reward at the end of the spur (Whittall & Hodges, 2007). Analysis of linkage disequilibrium in natural populations of *A. coerulea* in North America uncovered a QTL containing loss‐of function mutations in an *APETALA3‐3‐like* gene that causes petal‐like nectar spurs to be replaced with sepals (Cabin *et al*., 2022). This locus was under selection in areas with increased herbivory, suggesting a fitness advantage for these homeotic mutants. However, it also caused a shift in pollinator preference from hawkmoth to bumblebees, resulting in a decrease in outcrossing rates in populations containing the spurless morphology. This suggests that this locus is contributing to reproductive isolation in these *Aquilegia* populations, and it will be interesting to see whether these two floral morphologies lead to a speciation event in the future (Cabin *et al*., 2022). Together, these examples show how complex petal traits are evolving in response to changing environments, and highlights the importance of using foundational knowledge of developmental programmes to understand how they are altered in naturally evolving populations in real time.

## VIII. Conclusion and insights

Petals display various cellular arrangements that directly impact plant fitness, making them powerful systems to decipher how the interplay between evolutionary and developmental processes shapes the history of flowering plants. While significant progress has been made in understanding the genetic mechanisms and adaptive benefits of some of these cellular features like pigmentation patterns, other traits – such as cell shape variation, cuticle texture and the specification of various floral trichome types – require further characterization.

In addition, outstanding questions remain. For example, how such diverse cell types are specified within the same organ is far from understood. *MYB* transcription factors regulate tissue‐specific pigment production and, in some species, also influence cell shape or cuticle texture. However, how they regulate diverse traits across species and what controls their spatiotemporal expression remain elusive (Fig. 4). While recent studies in *Mimulus* and *Antirrhinum* hint at possible answers, such as the role of regulatory RNAs in controlling colour pattern production, whether this phenomenon applies across a broader range of species in not known. More importantly, upstream events that create subdomains within the petal primordia to allow neighbouring cells to embark on distinct growth and differentiation trajectories (Figs 4h, 6a) are still unknown, and solving this enigma constitutes an exciting challenge for the field.

Here, we argue that one way to answer some of these questions is by exploiting natural genetic and phenotypic variation across diverse species. The value of wild morphological variation has been highlighted in an increasing number of field studies and is certain to provide valuable insights into the vast diversity of flowering plants. Future research should focus on characterizing petal cell traits *in situ*, sequencing collections of novel ecotypes and developing robust transformation protocols for species well‐suited to investigate understudied petal features.

As we continue to unravel the genetic networks and key regulatory players of petal patterning, the integration of developmental genetics, genomics, high‐resolution imaging, mathematical modelling and ecological surveys will be crucial for advancing our understanding of petal patterning at the cellular level. We believe such interdisciplinary approaches are pivotal to move the field beyond descriptive studies to gain mechanistic insights on the complexity of patterning the microscale and its contribution to the evolution and diversification of flowering plants at the macroscale.

## Competing interests

None declared.

## Author contributions

ED and EM discussed ideas, prepared figures and wrote the manuscript.

## Disclaimer

The New Phytologist Foundation remains neutral with regard to jurisdictional claims in maps and in any institutional affiliations.
